# Development of a Polyolefin Elastomer Modified Hybrid Inorganic Filler System for Enhanced Performance in HDPE Double-Wall Corrugated Pipe Production

**DOI:** 10.3390/polym18030385

**Published:** 2026-01-31

**Authors:** Muhammet Ali Unal, Aysenur Sungur Bastug, Ece Yigit Ates, Ceyda Selcuk, Nisa Nur Ak, Recep Tolga Mutlu, Hilmi Saygin Sucuoglu, Bahadir Kaya

**Affiliations:** 1 Kuzey Boru A.Ş., R&D Center, Kırımlı Organized Industrial Zone, Aksaray 68100, Türkiye; aysenur.bastug@kuzeyboru.com.tr (A.S.B.); ece.ates@kuzeyboru.com.tr (E.Y.A.); ceyda.tekerbas@kuzeyboru.com.tr (C.S.); nisanur.ak@kuzeyboru.com.tr (N.N.A.); receptolga.mutlu@kuzeyboru.com.tr (R.T.M.); 2 Department of Mechanical Engineering, Aydin Adnan Menderes University, Aydin 09100, Türkiye; hilmisucuoglu@adu.edu.tr

**Keywords:** calcium carbonate, corrugated pipe, HDPE, inorganic filler, melt processing, polyolefin elastomer, polymer composites, talc

## Abstract

This study presents the design and performance evaluation of an advanced inorganic filler system composed of calcite (CaCO_3_) and talc (Mg_3_Si_4_O_10_(OH)_2_), modified with a polyolefin elastomer (POE), and integrated into a high-density polyethylene (HDPE) carrier resin with process additives such as erucamide, montan wax, pe wax, and PIB. The composite was developed to improve the structural integrity and longevity of HDPE double-wall corrugated pipes. Comprehensive characterization of the filler was performed using TGA–DSC, FTIR, SEM–EDX, XRD, and XRF analyses, confirming the presence of every individual component and homogeneous dispersion in the compound. Pilot-scale extrusion pipe trials confirmed uniform filler dispersion when evaluated by SEM-EDX analysis. The filler addition increased both the density and MFI values up to 1.03 g/cm^3^ and 1.5 g/10 min, respectively, while test results indicated oxidation induction times (OIT) reaching up to 40 min. The developed filler-added pipes demonstrated a significantly higher ring stiffness value of 12.20 kN/m^2^, exceeding the minimum requirement of 8 kN/m^2^ specified for the SN8 class pipes. The POE effectively attenuated rigidity and brittleness typically induced by mineral fillers, yielding this superior stiffness while maintaining adequate ring flexibility. These findings highlight the potential of this tailored filler system to advance the production of lightweight, mechanically robust corrugated piping solutions for demanding infrastructure applications.

## 1. Introduction

The incorporation of functional fillers is a critical strategy in polymer processing to reduce material costs, improve mechanical performance, and optimize rheological properties of polymer compounds [1,2,3,4]. Such fillers significantly enhance key material attributes, including elastic modulus, compressive and flexural strength, surface hardness, and thermal resistance. Moreover, specialized additive systems can impart additional functionalities such as antistatic behavior, flame retardancy, improved lubricity, and softening effects, thereby facilitating molding and extrusion processes [5]. One of the notable advantages of filler utilization is its ability to reduce polymer shrinkage during cooling, leading to improved dimensional stability and higher precision in molded components [6].

Among various fillers, calcium carbonate (CaCO_3_) is the most widely used due to its cost-effectiveness, stiffness enhancement effect, and positive influence on processability in extrusion-based manufacturing [7]. Typical formulations consist of 40–85 wt.% CaCO_3_, 15–60 wt.% polyethylene (HDPE, LLDPE, or LDPE) as the carrier matrix, and less than 3 wt% processing aids and slip agents [8]. While such systems are widely adopted, they often fail to meet the performance requirements of demanding applications. For example, in double-wall corrugated pipe production, high CaCO_3_ content can lead to flow instabilities, surface defects (e.g., dull appearance, sharkskin, and flow lines), reduced impact resistance, and decreased ring flexibility. These issues are further exacerbated by elevated extrusion pressures, which cause melt fracture, die build-up, and surface scratches, increasing scrap rates and production costs. To mitigate these limitations, some studies suggest that combining granular fillers with lamellar particles can offer synergistic benefits. For instance, talc (Mg_3_Si_4_O_10_(OH)_2_)—owing to its platy morphology—has been shown to improve processing lubricity and surface finish while enhancing the stiffness and thermal stability of the composite [9,10,11]. Therefore, a hybrid filler system combining CaCO_3_ and Talc presents a promising approach to balance cost with the rigorous mechanical demands of structural applications.

The effectiveness of such high-fill systems is fundamentally dependent on the polymer matrix and interfacial modifiers. While mineral-filled polyolefin systems have been widely studied, most research has focused on using either micron-sized calcium carbonate or talc as single fillers in polyethylene matrices. However, these approaches often suffer from limited filler dispersion, weak interfacial bonding and reduced ductility, particularly in pipeline applications. While these filler systems have been extensively investigated in general polymer compounds, their translation to double-wall corrugated pipe extrusion—where rheological stability, ring stiffness, etc. must be simultaneously satisfied—remains insufficiently addressed. This study addresses these issues by developing a multi-filler system modified with carrier resin and a polyolefin elastomer (POE) specifically designed for HDPE double-wall corrugated pipes. High Density Polyethylene (HDPE) is the preferred carrier resin due to its superior tensile strength and chemical resistance, which supports the structural integrity of masterbatch compounds [12,13,14,15]. However, to counteract the brittleness induced by high mineral loading, the inclusion of impact modifiers is essential. Polyolefin elastomers (POEs), synthesized via metallocene catalysis, have demonstrated a unique ability to increase ductility and impact strength without significantly compromising the stiffness of the various polymer matrix systems [16,17,18]. Furthermore, the processability of these complex formulations relies heavily on specialized additive systems. Research indicates that the combination of lubricants (e.g., waxes, stearates) and dispersion aids like Polyisobutylene (PIB) significantly reduces metal–polymer interfacial friction and stabilizes extrusion pressure, thereby minimizing surface roughness in highly filled compounds [19,20,21,22,23].

Consequently, the main objective of this work is to decrease the scrap rate in pipe production and produce mechanically durable pipes through single-screw extrusion, while minimizing material waste and improving manufacturing efficiency. The proposed granular filler combines optimally balanced ratios of calcium carbonate (CaCO_3_) and talc within an HDPE carrier resin and POE to enhance flexibility and ring stiffness. This work provides a practical and scalable filler design that improves processability and mechanical reliability in industrial extrusion, supporting the development of more durable and sustainable piping materials. The chemical, morphological, and mechanical characteristics of the filler system, as well as its performance under industrial-scale extrusion conditions, are comprehensively examined. This study is not intended as a parametric optimization of filler compositions, but rather as a design-driven investigation of a specifically engineered hybrid inorganic filler system tailored for industrial double-wall corrugated pipe extrusion. The focus is placed on demonstrating process stability, dispersion quality, and mechanical performance under realistic single-screw extrusion conditions, which are often overlooked in laboratory-scale parametric studies.

## 2. Materials and Methods

### 2.1. Materials

The composite filler formulation was prepared using inorganic mineral fillers and a polyolefin-based carrier resin. Calcium carbonate (CaCO_3_) (average particle size: 3.3 μm, purity: 99%) and talc (Mg_3_Si_4_O_10_(OH)_2_) (average particle size: 4.1 μm, lamellar structure, purity: 99–100%) were selected as primary fillers to enhance stiffness and dimensional stability. High density polyethylene (HDPE) (MFI: 1.2 g/10 min, density: 0.954 g/cm^3^) was used as the carrier resin. Polyolefin elastomer (POE) (MFI: 1.0 g/10 min, density: 0.868 g/cm^3^) was incorporated as an impact modifier (Table 1).

Processing additives included erucamide (refined, melting point:83 ± 5 °C) as an internal slip agent, polyethylene (PE) wax (density: 0.96–0.98 g/cm^3^, drop point: 127–132 °C) and esterified montan wax (drop point: 99–105 °C, acid value < 15 mg KOH/g) as external lubricants, and polyisobutylene (PIB, density:0.867 g/cm^3^ at 15 °C) as an auxiliary processing oil. All raw materials were used without further purification.

The components of the filler material were blended using the pre-mix method prior to extrusion. All raw materials were mixed until a homogeneous blend was obtained. After the mixing process, the mixture was transferred to the extrusion lines for further granulation.

This study is based on a novel composite formulation developed by Kuzey Boru R&D Center. The overall composition of the filler system was formulated as 80 wt.% inorganic components, 18 wt.% thermoplastics (HDPE/POE), and 2 wt.% processing additives (erucamide, montan wax, pe wax, PIB), enabling both reliable laboratory evaluation and applicability to pipe manufacturing processes.

### 2.2. Compounding of Filler Masterbatch

The filler masterbatch was prepared using a co-rotating twin-screw extruder (Shanghai Jwell Machinery Co., Ltd., Shanghai, China; screw diameter: 135 mm, L/D ratio: 30:1) equipped with a gravimetric feeder. The temperature profile was set between 60 and 200 °C, and the screw speed at 25–30 rpm.

### 2.3. Production of Corrugated Pipes

Double-wall corrugated pipes with a nominal diameter of 200 mm, SN8 class, were manufactured using a co-extrusion corrugated molding system. The inner wall was extruded from 73 wt.% neat HDPE (Sabic^®^ HDPE, Riyadh, Saudi Arabia, MFI: 0.26 g/10 min, density: 0.958 g/cm^3^), 25 wt.% filler masterbatch compound and 2 wt.% black colorant (Maskom, Düzce, Türkiye) MFI: 0.5 g/10 min, density: 1.13 g/cm^3^) and the outer wall from 50 wt.% neat HDPE (Sabic^®^ HDPE, MFI: 0.26 g/10 min, density: 0.958 g/cm^3^), 48 wt.% filler masterbatch compound and 2 wt.% yellow colorant (Maskom, Düzce, Türkiye, MFI: 2.4 g/10 min, density: 0.944 g/cm^3^). A dosing system was employed to feed both the main and co-extruders. The melt temperature was 190–195 °C, and vacuum-assisted forming ensured dimensional accuracy. Pipes were produced according to ISO 13476-3 [24].

**Figure 1:**
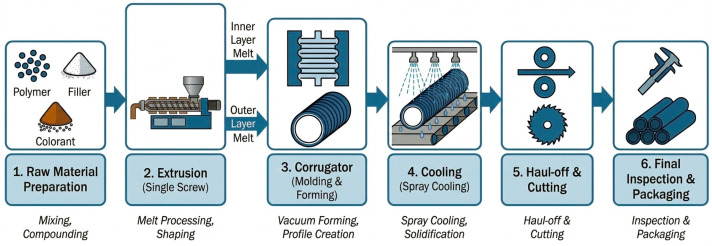
Schematic flowchart of the double-wall corrugated pipe manufacturing procedure.

Double-wall corrugated pipes were produced using a co-extrusion line equipped with two single-screw extruders (Shanghai Jwell Machinery Co., Ltd., Shanghai, China): Main extruder (outer wall): 60 mm screw diameter, L/D ratio 36:1; Co-extruder (inner wall): 60 mm screw diameter, L/D ratio 36:1. The corrugator speed was 1.5 m/min (schematized in Figure 1).

**Figure 2:**
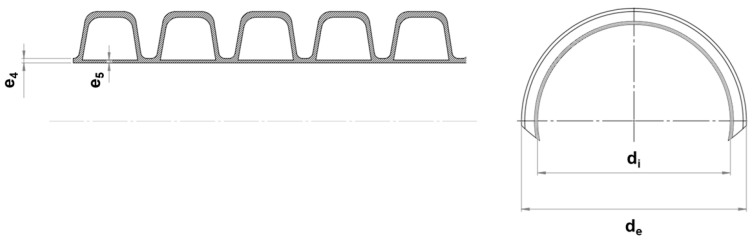
Schematic representation of the cross-section side of a corrugated pipe (e_4_: wall thickness of the inside layer; e_5_: wall thickness of the inside layer under a hollow section; d_i_: inside diameter; d_e_: outside diameter).

**Figure 3:**
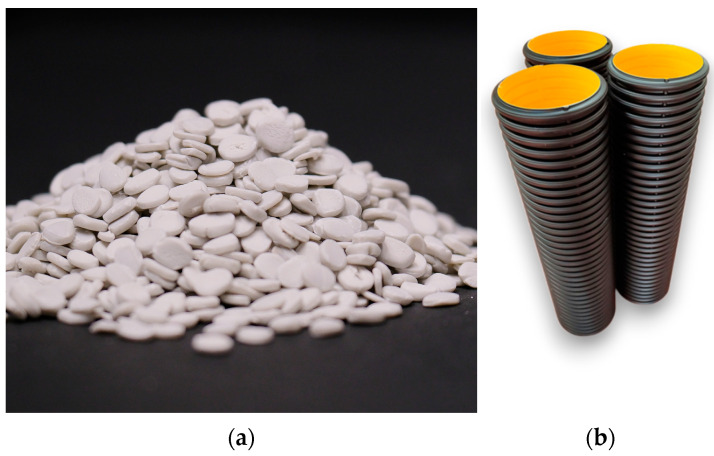
(**a**) Image of the POE-modified hybrid CaCO_3_/Talc filler system in granular form; (**b**) ∅200 HDPE double-wall corrugated pipes produced with the POE-modified hybrid filler system.

Both extruders were controlled independently with temperature zones as follows: for the main extruder, 80–190 °C with a screw speed of 65 rpm, and for the co-extruder, 80–185 °C with a screw speed of 44 rpm. Schematic representative of the cross-section of corrugated pipe is shown in Figure 2 and Table 2. In addition, images of the hybrid filler and manufactured test pipes are given in Figure 3.

### 2.4. Characterization

#### 2.4.1. X-ray Diffraction (XRD) Measurement

X-ray Diffraction tests were performed using a Rigaku Miniflex 600 model diffractometer (Rigaku Corporation, Tokyo, Japan) using Cu-Ka radiation at 40 kV and 15 mA. XRD patterns were acquired in the 2θ range from 5° to 80° with 0.02° s^−1^ per step.

#### 2.4.2. X-ray Fluorescence (XRF) Analysis

The elemental composition of the samples was determined using X-ray fluorescence (XRF) spectroscopy. The analysis was performed with a Rigaku ZSX Primus model XRF spectrometer (Rigaku Corporation, Tokyo, Japan).

#### 2.4.3. Morphological Analysis

The micrographs were taken with a Zeiss Supra 50 VPTM model scanning electron microscope (Carl Zeiss Microscopy GmbH, Jena, Germany) and samples were analyzed by energy dispersive X-ray spectroscopy (EDS) using a Si/Li detector fitted to the microscope. The images were generated from secondary electrons and to avoid surface charging the material was coated with a thin layer of gold.

#### 2.4.4. Thermal Analysis

Thermal characterization of the samples was simultaneously performed by Differential Scanning Calorimetry (DSC) and Thermogravimetry Differential Thermal analysis (TG/DTA) analyses using a NETZSCH STA 449F3 (NETZSCH-Gerätebau GmbH, Selb, Germany) instrument. Samples were heated from 25 °C to 1250 °C. The heating and cooling rates of 10 °C/min under a nitrogen atmosphere were used during the test.

#### 2.4.5. FT-IR Spectrum Measurement

The FT-IR spectra were recorded using a Thermo Scientific Nicolet iS10 FTIR spectrophotometer (Thermo Fisher Scientific Inc., Waltham, MA, USA) over the spectral range of 400–4000 cm^−1^.

#### 2.4.6. Density Determination

The density (ρ) of the test specimens was determined according to the ISO 1183-1:2019 [25] with the following equation:

(1) $$\rho =\left[\frac{W_{1}}{W_{1}-W_{2}}\right]\times \rho_{W}$$

where $W_{1}$ and $W_{2}$ are the sample weights in air and ethanol, respectively, and $\rho_{W}$ is the density of ethanol.

#### 2.4.7. Ash Content Determination

The inorganic residue of the samples was determined according to ISO 3451-1 [26]. The specimens were subjected to calcination at 900 °C in a laboratory muffle furnace until a constant mass was achieved. The ash content provides a quantitative measure of the non-combustible fillers and additives present in the formulation.

#### 2.4.8. Melt Flow Index (MFI) Analysis

The melt flow rate (MFI) of the samples was determined in accordance with ISO 1133-1 [27]. The measurements were conducted using a standard melt flow indexer at a test load of 5 kg and a temperature of 190 °C. This method was employed to evaluate the processability and flow characteristics of polymeric materials.

#### 2.4.9. Oxidation Induction Time (OIT) Test

The oxidation induction time was determined using Differential Scanning Calorimetry (DSC) in accordance with ISO 11357-6 [28]. The procedure followed the guidelines of ISO 11357-1 [29] with Indium as the reference material for instrument calibration. This test was conducted to assess the oxidative stability of the polymer matrix.

#### 2.4.10. Ring Stiffness Test

**Figure 4:**
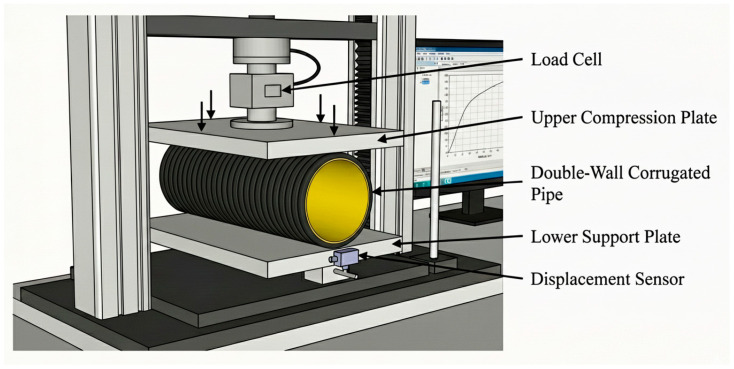
Experimental setup for ring stiffness and ring flexibility tests.

The ring stiffness of the samples was evaluated following ISO 9969 [30]. Specimens were tested under a controlled compressive load at a crosshead speed of 5 mm/min. This method provides a quantitative measure of the pipe’s resistance to external loading and ovalization (schematized in Figure 4).

(2) $$S=\left(0.0186+0.025\frac{y}{d}\right)\frac{F}{Ly}10^{6}$$

*S*: Ring Stiffness (kN/m^2^)

*F*: Loading force (kN)

*L*: Length of the test piece (mm)

*y*: Vertical deflection (mm)

*d_i_*: Inside diameter of the test piece of pipe (mm)

#### 2.4.11. Ring Flexibility Test

Ring flexibility tests were performed in compliance with ISO 13968 [31] to assess the deformation resistance of the pipe specimens under bending stresses. The experiments were carried out at a crosshead speed of 5 mm/min, as specified by the standard, to ensure consistent loading conditions.

## 3. Results & Discussion

### 3.1. Characterization of the Filler

#### 3.1.1. XRD & XRF Results

**Figure 5:**
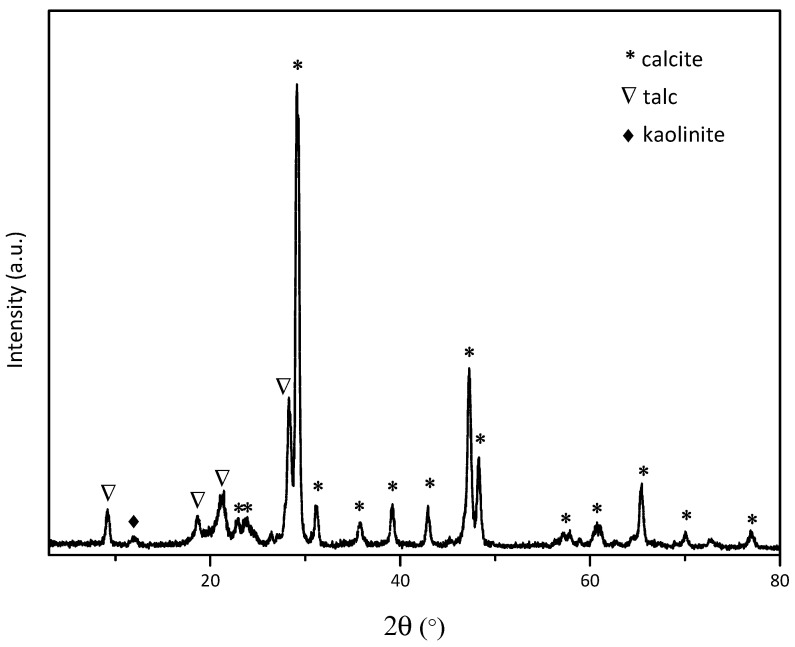
X-ray diffraction pattern of composite filler.

The bulk compositions of the filler as determined by XRF are shown in Table 3, including measured concentrations and detection limits for each element. Quantitative mineralogical analysis of the filler also is presented in Table 4. The X-ray diffraction pattern of the composite filler in Figure 5 shows the characteristic peak distribution of calcite, which is a major phase, 76.8 wt.%, and talc, which is secondary phase, 21.7 wt.% according to XRF results. Minor peaks corresponding to kaolinite, 1.5 wt.%, were also identified, indicating the presence of a small impurity phase in the compound. Although the filler composite contains HDPE and POE as the polymeric phases, no detectable crystalline peaks of these polymers were observed in the XRD pattern. This can be attributed to I) the relatively low content of the polymers compared to the dominant mineral phases, II) the semi-crystalline or amorphous nature of the HDPE carrier resin and POE component, and III) the limited scattering contrast of low-Z elements in XRD analysis [32].

These results confirm that the inorganic fraction of the composite is dominated by calcite and talc, with kaolinite present as a minor impurity, while the polymeric components exist in a matrix phase but do not contribute significant diffraction peaks under the measurement conditions.

#### 3.1.2. Morphological Analysis

**Figure 6:**
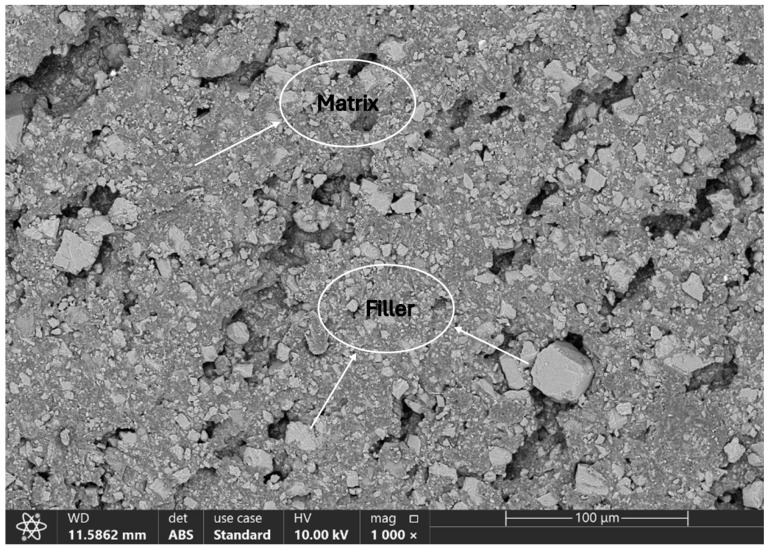
SEM micrograph of inorganic composite filler.

**Figure A1:**
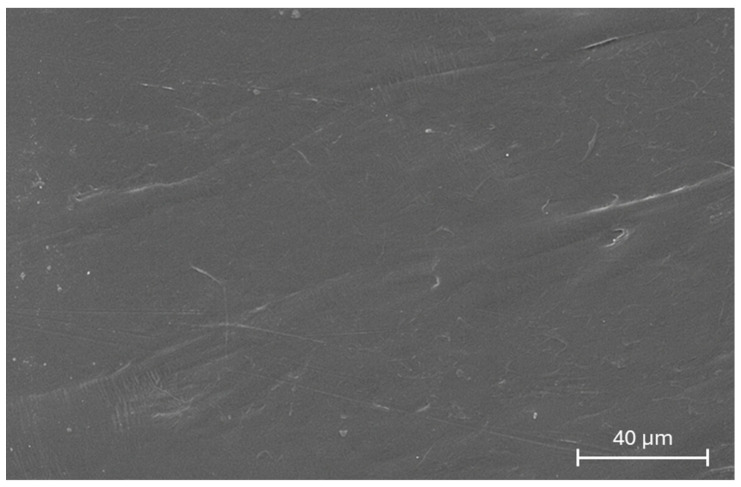
SEM micrograph of Neat HDPE.

In Figure 6, the SEM image of the hybrid filler clearly shows the finely dispersed and heterogeneous microstructure typical of a multi-component system. Distinct mineral phases of plate-like talc and angular calcite are easily recognized in the image. Other components, such as HDPE (as shown in Figure A1) and POE polymeric phases, appear as smooth, continuous regions surrounding or bridging the mineral particles, suggesting interfacial compatibility between the inorganic and organic components. Although the process additives cannot be directly visualized due to their small size and low contrast, their presence can be inferred from the uniform distribution of the mineral and polymer phases, consistent with their role in facilitating dispersion and modifying the melt rheology during compound processing [33].

**Figure 7:**
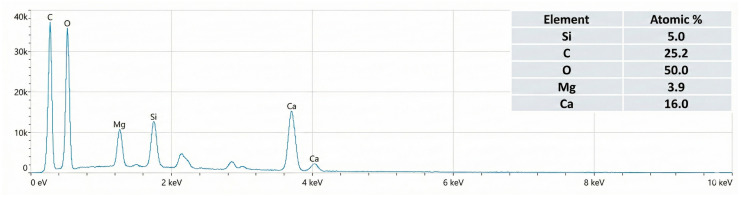
Representative EDX spectrum confirming elemental analysis of hybrid filler.

The accompanying EDX spectrum corroborates the compositional identity of the filler (Figure 7). Prominent Ca and O peaks confirm the presence of calcium carbonate, whereas Si and Mg signals are characteristic of talc. The intense C signal arises from the HDPE carrier resin and POE as well as carbon-based process additives. As expected, H and N coming from erucamide, montan wax, and other organics are absent from the spectrum because the EDX detector cannot reliably detect low-atomic-number elements.

**Figure 8:**
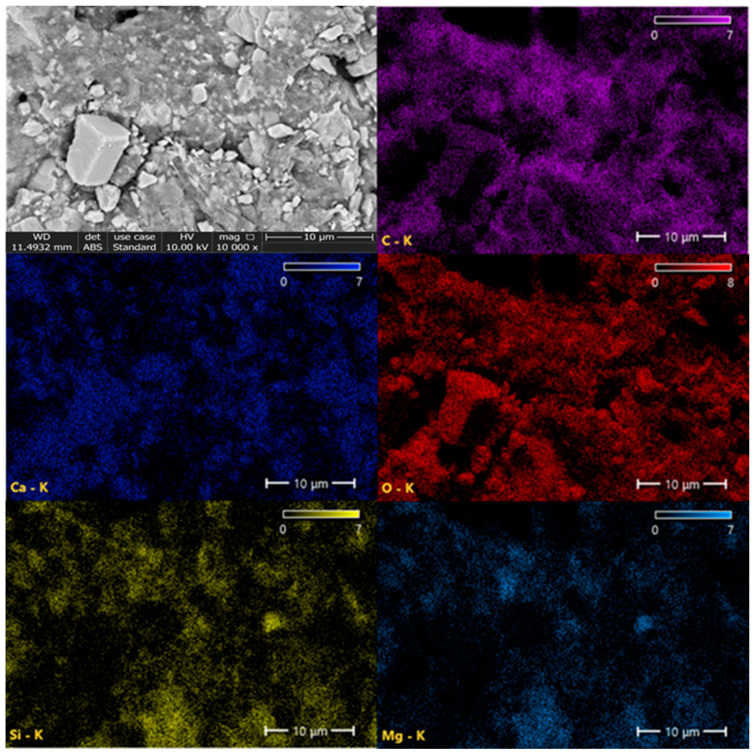
SEM electron image and elemental signal maps of the filler (Detector: ABS, Acceleration Voltage: 10 kV, C: carbon, Ca: calcium, O: oxygen, Si: Silicon, Mg: magnesium, Maps Resolution: 768 × 512).

**Figure A2:**
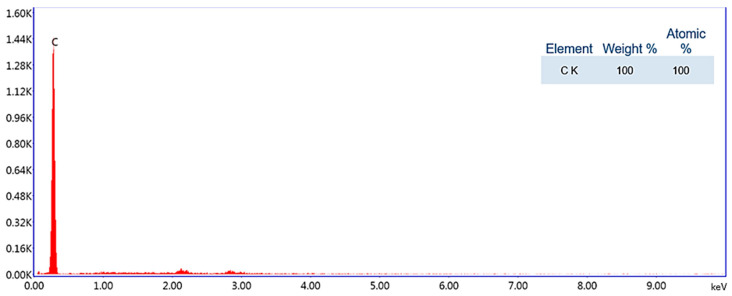
Representative EDX spectrum confirming elemental analysis of Neat HDPE.

Elemental mapping demonstrates a uniform distribution of all detected elements throughout the filler granule (Figure 8). Ca and O are co-localized in the calcite-rich domains, while Si and Mg are concentrated in talc platelets, confirming the preservation of individual mineral phases. Carbon mapping shows continuous coverage of the polymeric carrier resin HDPE (as shown in Figure A2), and the component of POE are well-integrated with mineral particles and evidence of carbonate structure (-CO_3_^2−^). This homogeneous dispersion is critical for achieving consistent mechanical, thermal, and rheological properties in the co-extruded pipe walls.

Overall, the SEM and EDX analyses provide clear evidence that the hybrid filler is well dispersed, with close contact between mineral and polymeric phases, which supports the observed improvements in melt flow, density, oxidative stability, and mechanical performance of the final HDPE pipe compounds.

**Figure 9:**
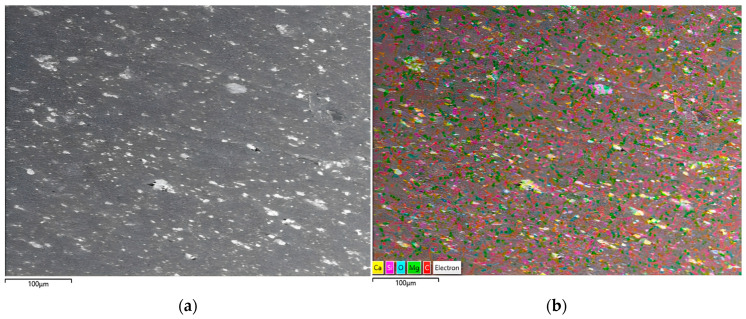
(**a**) SEM micrograph of a corrugated pipe cut surface; (**b**) elemental mapping image of corrugated pipe cut surface (Detector: BS, Acceleration Voltage: 15 kV, C: carbon (Red), Ca: calcium (Yellow), O: oxygen (Blue), Si: Silicon (Purple), Mg: magnesium (Green)).

Figure 9 illustrates the SEM image obtained from the cross-section of the corrugated pipe along with its corresponding elemental mapping analysis, confirming a homogeneous distribution of the filler material within the HDPE polymer matrix.

#### 3.1.3. Thermal Analysis

**Figure 10:**
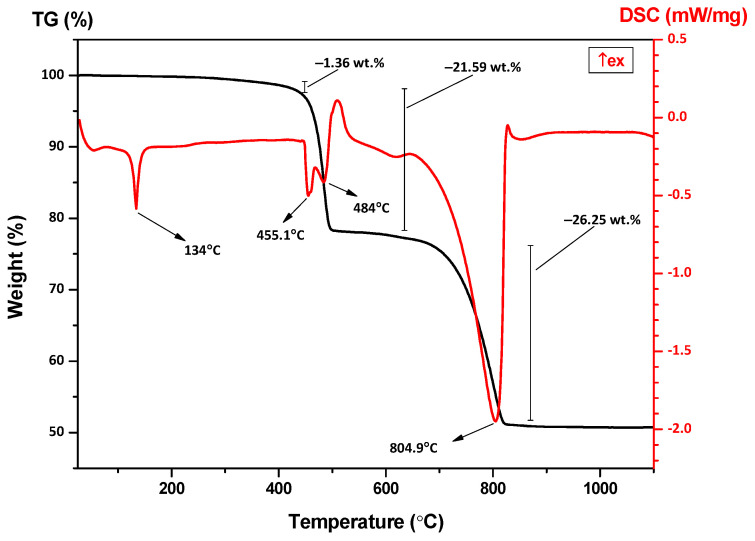
TG-DSC analyses of the filler component.

The TG curve shows a minor mass loss of approximately 1.36 wt.% was observed up to 200–250 °C, accompanied by an endothermic deviation in the DSC profiles (Figure 10). This phenomenon is ascribed to the volatilization of physically adsorbed moisture and polymeric additives. A DSC endothermic peak at 134 °C corresponds to crystalline melting of HDPE carrier resin, modified by filler presence.

**Figure A3:**
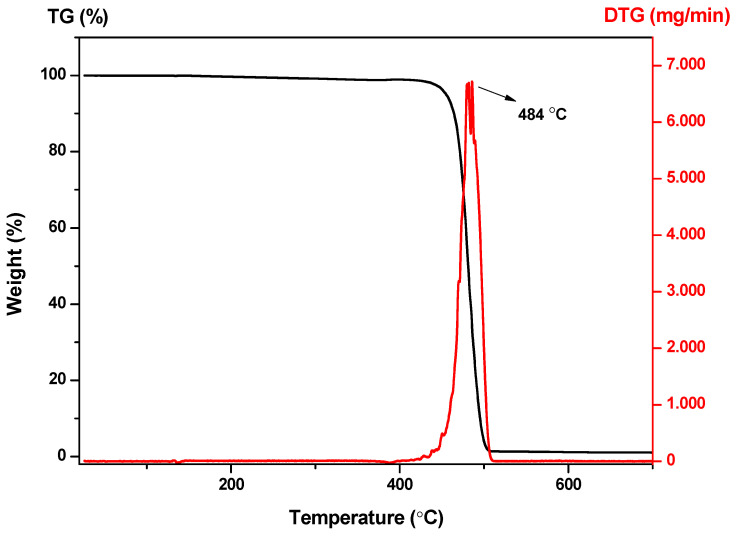
TGA analysis of Neat HDPE.

A pronounced mass loss with distinct TG peak is detected in the 450–500 °C range, which corresponds to the thermal degradation of the HDPE carrier resin and the polyolefin elastomer phase. The occurrence of overlapping peaks (at 455.1 °C and 484 °C, endothermic under inert conditions) suggests multiple degradation pathways, likely attributable to bulk HDPE (as shown in Figure A3) chains and the elastomeric component, with possible contributions from internal sliding agents or waxes that may reduce thermal stability or generate secondary decomposition features [34,35].

At higher temperatures (around 800–805 °C), a major mass loss of approximately 26% is recorded together with a sharp DSC endothermic peak at 801 °C, corresponding to the thermal decomposition of CaCO_3_ (calcite) into CaO and CO_2_. This decarbonation process, well documented for calcite-filled composite structures, typically initiates above 700–750 °C under heating, depending on particle size, surface area, and dispersion state [36].

The TG-DSC data do not show a distinct decomposition event assignable to talc (Mg_3_Si_4_O_10_(OH)_2_) in the range up to 1100 °C. Talc is expected to dehydroxylate (i.e., lose structural hydroxyl groups) and decompose at higher temperatures. In the literature, onset of talc decomposition is around 800 °C, with major dihydroxylation peaking at 895 °C [37]. The thermogravimetric signal corresponding to talc dehydroxylation may be challenging to isolate because of the substantial mass loss attributed to the calcium carbonate decomposition and/or its potential alteration through organic-filler interactions. Also, if the talc used is of high purity, well dehydrated, or surface treated, its dehydroxylation may be delayed or its signal damped. The large endothermic peak near 801 °C in DSC curve matches the calcite decomposition observed by TG analysis, providing consistency between mass loss and heat flow. The presence of internal/external lubricants and waxes, along with the existing polyolefin elastomer component, may result in multiple, overlapping decomposition events.

#### 3.1.4. FT-IR Spectroscopy

**Figure 11:**
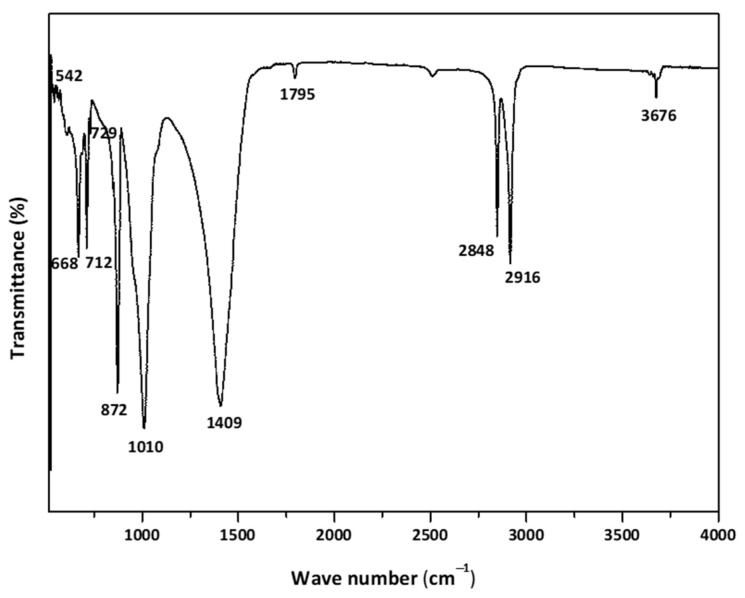
FT-IR spectra of the filler material.

The presence of chemical bonds within the filler’s components was studied using FT-IR spectroscopy. The FTIR spectra of the filler is shown in Figure 11. The spectrum reveals several distinct absorption bands, each corresponding to a specific vibrational mode of the constituent molecules.

**Figure A4:**
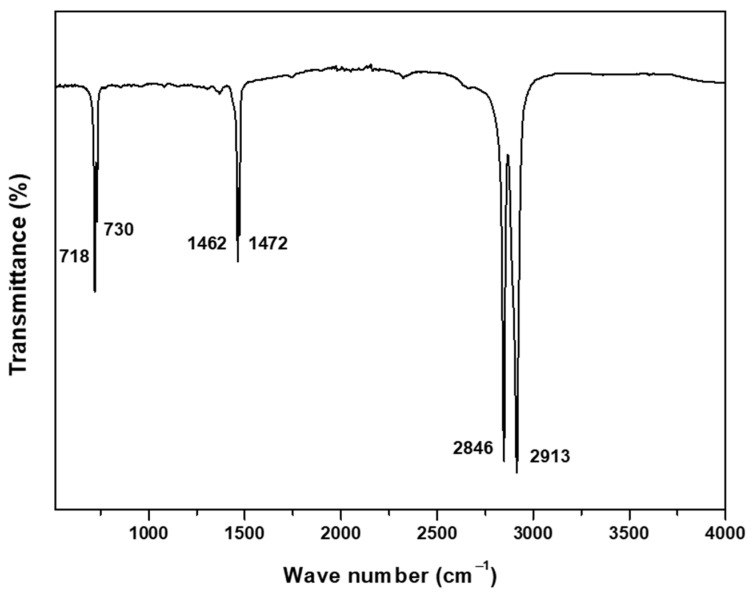
FT-IR Spectra of the Neat HDPE.

The strong bands at 2916 cm^−1^ and 2848 cm^−1^ correspond to the asymmetric and symmetric CH_2_ stretching vibrations of the polyethylene backbone (as shown in Figure A4) and the polyolefin elastomer, with additional contributions from the long-chain aliphatic lubricants (erucamide, montan wax, PE wax, PIB) [38,39,40,41]. A weak but distinct band at 3676 cm^−1^ is attributed to structural hydroxyl groups in talc (Mg_3_Si_4_O_10_(OH)_2_) or residual surface hydroxyls from filler treatments [42].

A minor band near 1795 cm^−1^ can be ascribed to carbonyl functionalities present in fatty-acid amide slip agents (erucamide) or montan wax and may also reflect minor oxidative products in the polyolefin phase. Although erucamide, montan wax, and polyisobutylene exhibit characteristic FT-IR absorptions at 3320 cm^−1^ and 1640 cm^−1^ (N-H and C=O of erucamide), 1735 cm^−1^ (C=O of montan wax), and 1365–1390 cm^−1^ (CH deformation of PIB), no distinct peaks at these positions are discernible in the present spectra. This absence is most likely due to the very low concentration of these additives in the composite (≤3 wt.% in the w/o), which results in weak IR absorptions that are further masked by the intense and overlapping CH_2_ vibrations of the polyolefin.

In the fingerprint region, the broad absorption around 1460–1409 cm^−1^ arises from a combination of CH_2_ bending vibrations of the polyolefin chains and the asymmetric stretching of carbonate groups (-CO_3_^2−^) from calcite [43]. Diagnostic calcite peaks are also observed at 872 cm^−1^ (-CO_3_^2−^ out of plane bending) and 712 cm^−1^ (-CO_3_^2−^ in plane bending) [44].

The band at 1010 cm^−1^ is typical of Si-O stretching in talc, while features at 686 cm^−1^ and 542 cm^−1^ correspond to Si-O-Mg lattice vibrations and deformation modes of the silicate filler [45]. The multiple peaks in the 729–542 cm^−1^ region also reflect overlapping CH_2_ rocking modes of the polyolefin matrix.

The assignments listed in Table 5 according to the literature clearly demonstrate the coexistence of the mineral fillers (calcite and talc), the polyolefin elastomer, waxy/lubricant additives, and polyethylene as a carrier resin, thereby confirming the targeted multi-component formulation and supporting the interpretations drawn from the TG-DSC/XRD-XRF/SEM-EDX results. The comprehensive FT-IR analysis provides conclusive evidence for the presence of the main specified components in the filler, with characteristic absorption bands of calcite, talc, HDPE, the polyolefin elastomer, and the organic additives (erucamide, montan wax, PE wax, and PIB) identified.

### 3.2. Testing and Analysis of HDPE Double-Wall Corrugated Pipes Incorporating a Novel Filler

#### 3.2.1. Density and Ash Content Determination

**Figure 12:**
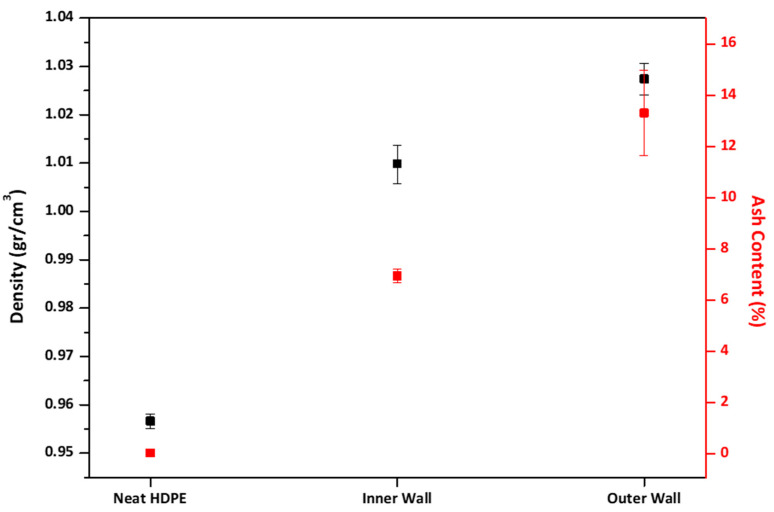
Comparison of the density and ash content results of Neat HDPE resin and corrugated pipe walls.

The density and ash content results reveal the influence of mineral based filler incorporation on the corrugated pipe walls in Figure 12. Neat HDPE displayed a narrow density range of 0.955–0.958 g/cm^−3^, typical for high molecular weight semi-crystalline HDPE resins. In contrast, the inner wall exhibited higher densities (1.006–1.015 g/cm^−3^), and the outer wall reached similar ranges (1.024–1.032 g/cm^−3^). This density increases correlates with the higher specific gravity of the filler (density of the filler is measured at 1.87 g/cm^−3^) compared with the polymer matrix HDPE. Filler content increases directly with composite density, which offers a simple way to estimate the loading levels and dispersion.

Ash content measurements provide corroboration for this interpretation. The neat HDPE material contained only a trace amount of inorganic residue (0.03–0.05%), which is consistent with the standard pigment levels found in commercial-grade resins. Conversely, the inner wall exhibited ash contents of 6.53–7.14%, while the outer wall reached a considerably higher range of 12.43–15.80%. These gravimetric results align quantitatively with the designed formulation ratios (25 wt.% filler in the inner wall and 48 wt.% filler in the outer wall), after accounting for the non-combustible components within the polymeric carrier and waxy processing additives. These values are suitable according to ISO 13476-3, which states that ash residue should not exceed 25% in the pipe. Significantly, the consistency and low variability of the ash result across multiple replicates suggest uniform filler incorporation and effective process control, both of which are critical determinants of mechanical performance and long-term durability [46].

#### 3.2.2. Melt Flow Index (MFI) Analysis

**Figure 13:**
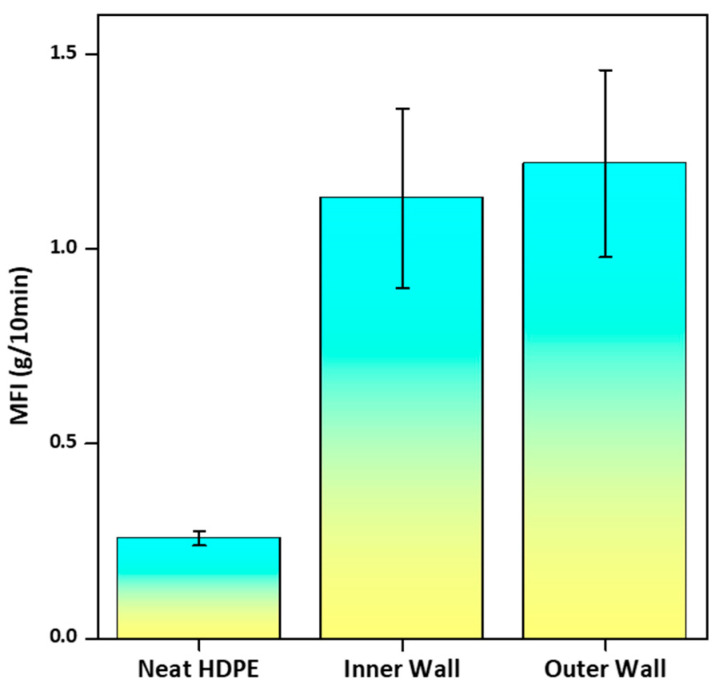
Comparison of the MFI values between the Neat HDPE resin and corrugated pipe walls.

The MFI data revealed clear differences between the Neat HDPE, the inner wall, and the outer wall (Figure 13). While neat HDPE exhibited a low MFI (0.24–0.28 g/10 min), the inner wall showed higher values (0.94–1.4 g/10 min), and the outer wall exhibited a similar range (1–1.5 g/10 min). This behavior arises from the combined effects of mineral fillers, polymeric carrier phases, and processing additives. Although mineral fillers generally restrict polymer chain mobility and increase melt viscosity, talc is known to promote shear-induced slip and platelet alignment, which can reduce flow resistance and increase MFI in polyethylene systems [3]. Furthermore, the incorporation of high filler loadings alters the melt stress distribution during extrusion, which may partially disrupt polymer chain packing and contribute to higher apparent MFI values [47], without indicating severe degradation.

Processing additives play a critical role in amplifying this effect. Polyethylene wax and esterified montan wax act as low-molecular-weight external lubricants, improving filler dispersion, reducing interparticle friction, and lowering the apparent melt viscosity. Erucamide further reduces die-wall friction through interfacial migration during extrusion, enhancing melt slip and stabilizing flow. Together, these additives act synergistically with the POE and HDPE as a carrier resin to counterbalance the inherent viscosity increase from fillers. It is clear that the MFI variation in highly filled HDPE pipe materials is a complex issue. The MFI is not just a function of the inorganic filler amount; it is a consequence of the simultaneous effects that additives (like polymeric carriers, wax lubricants, and slip agents) have on the molten polymer’s structure and flow properties during processing.

#### 3.2.3. Oxidation Induction Time (OIT) Test

**Figure 14:**
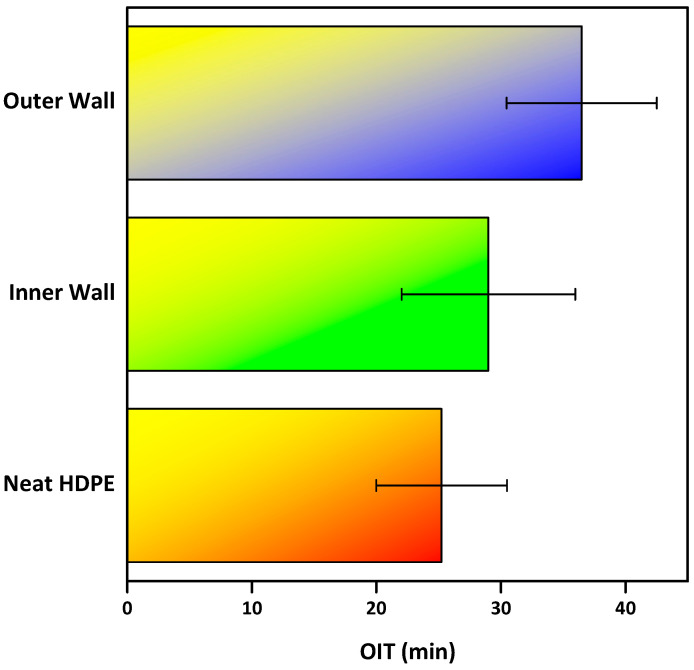
OIT test values between the Neat HDPE resin and corrugated pipe walls.

The oxidation induction time (OIT) results provide insight into the thermal-oxidative stability of the neat resin and the co-extruded walls (Figure 14). The Neat HDPE exhibited OIT values ranging from 22 to 33 min., reflecting variability in the baseline antioxidant content of the commercial SABIC grade. In comparison, the inner wall showed more consistent OIT values of 32–37 min, while the outer wall ranged from 30 to 43 min. These results indicate that the compounded pipe walls retain or even enhance oxidative stability relative to the neat resin despite significant mineral loading.

**Figure 15:**
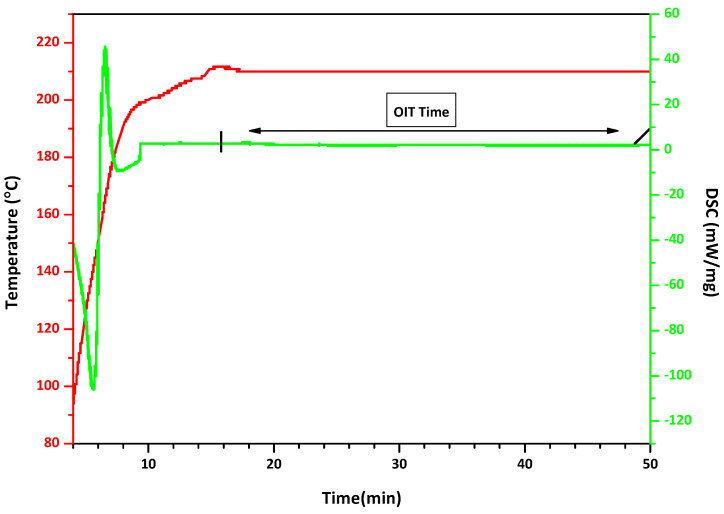
An example of OIT test result attained from a corrugated pipe test sample.

This behavior can be attributed to the presence of mineral fillers such as calcium carbonate and talc, as they limit the polymer exposure to oxygen and heat, enhance thermal stability, and contribute to flame retardancy by reducing polymer flammability [48,49,50]. This behavior is primarily attributed to the barrier and dilution effects introduced by inorganic fillers such as calcium carbonate and talc. The mineral phases reduce the effective polymer volume fraction exposed to oxygen and heat, thereby slowing oxidation kinetics. Maintaining OIT values above 20 min is generally regarded as indicative of adequate antioxidant protection for long-term service in HDPE pipes (Figure 15). These findings confirm that, when properly formulated, highly filled HDPE pipe compounds can achieve not only the desired stiffness and processability but also the oxidative stability required for durable performance.

#### 3.2.4. Ring Stiffness and Ring Flexibility Tests

**Figure 16:**
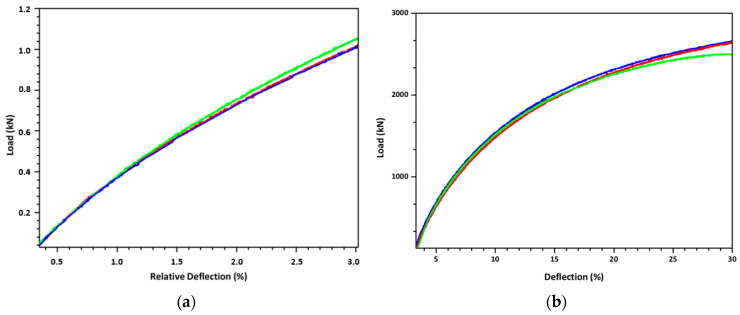
(**a**) Ring stiffness and (**b**) ring flexibility graphs of tested ∅200 SN8 class HDPE double-wall corrugated pipes reinforced with hybrid filler (three specimens). The green, red, and blue curves correspond to Specimen 1, Specimen 2, and Specimen 3, respectively.

The ring stiffness and ring flexibility test results are shown in Figure 16. The ring stiffness test (Figure 16a) evaluates the initial structural rigidity of the pipe at low deformation levels (~3%), whereas the ring flexibility test (Figure 16b) assesses the ability of the pipe to sustain large deformations (up to 30%) without structural failure. While ring stiffness reflects load-bearing capacity, ring flexibility highlights ductility and interlayer integrity, and the results show no significant loss of flexibility despite increased stiffness. The filler-added pipes exhibited ring stiffness values between 9.43 and 12.20 kN/m^2^ (mean nearly at 10.7 kN/m^2^), exceeding the minimum requirement of 8 kN/m^2^ specified for the SN8 class pipes. This corresponds to an approximate 30–35% increase in average stiffness with the new formulation. Ring stiffness is a key parameter in corrugated pipe performance, directly reflecting the composite’s resistance to external loads and long-term deformation [51].

The observed improvement consists of the higher mineral content and optimized filler system used in the new designed filler formulation. Increasing the proportion of rigid inorganic fillers, particularly calcium carbonate and talc, increases the composite modulus and enhances the load-bearing capacity of the corrugated structure. At the same time, the addition of a polymeric HDPE carrier resin and polyolefin elastomer (POE) in the filler improves dispersion and interfacial bonding between the filler granules and HDPE matrix resin, reducing stress concentrations that often cause premature buckling of the pipe. Processing aids such as polyethylene wax, montan wax, and erucamide further contribute by lowering melt viscosity during extrusion, promoting more uniform wall thickness and consistent corrugation geometry. This combination results in pipes with a denser, more homogeneous microstructure and higher stiffness without compromising processability.

Notably, the formulation increases ring stiffness while maintaining adequate melt flow, density, and oxidative stability, as shown earlier. This demonstrates the advantage of balancing inorganic filler type, carrier resin, and processing additives to improve both stiffness and durability. Such an integrated approach aligns with trends in high-performance polyolefin pipe technology, combining cost-effective mineral loading with tailored rheology and stabilization systems to meet strict mechanical and longevity standards.

**Figure 17:**
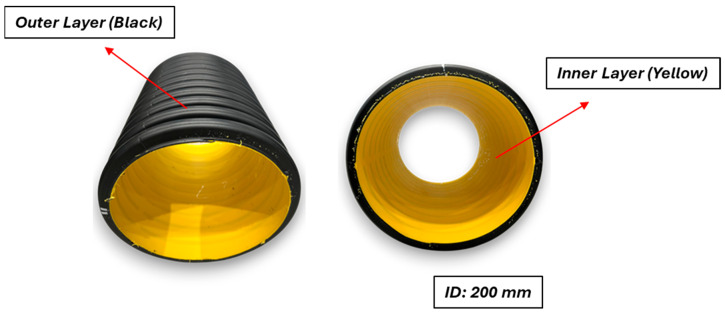
Image of ∅200 HDPE double wall corrugated pipes after ring flexibility test according to relevant standard test method (ID: Inner Diameter).

Ring flexibility testing showed that all evaluated pipes maintained structural integrity under the specified deformation, with no visible cracking, delamination or wall collapse (Figure 17). The new filler-enhanced HDPE pipes withstood the required deflection without damage, indicating that high mineral loading and the modified formulation did not reduce ductility or interwall adhesion. This performance is crucial for buried applications, where pipes endure repeated soil loads and deflections during service.

The lack of damage at the specified deflection indicates that the combined use of HDPE carrier resin, POE, and erucamide/wax-type processing aids effectively preserved the matrix’s toughness and interfacial cohesion despite the increased stiffness from the inorganics such as calcite and talc. Maintaining ring flexibility alongside high stiffness demonstrates a well-balanced formulation, as excessive filler loading or poor dispersion usually causes embrittlement and crack initiation under flexural strain. These results confirm that the developed compound meets the dual requirements of stiffness and flexibility outlined in standards for corrugated pipes, ensuring both load-bearing capacity and deformation tolerance during installation and service.

**Figure 18:**
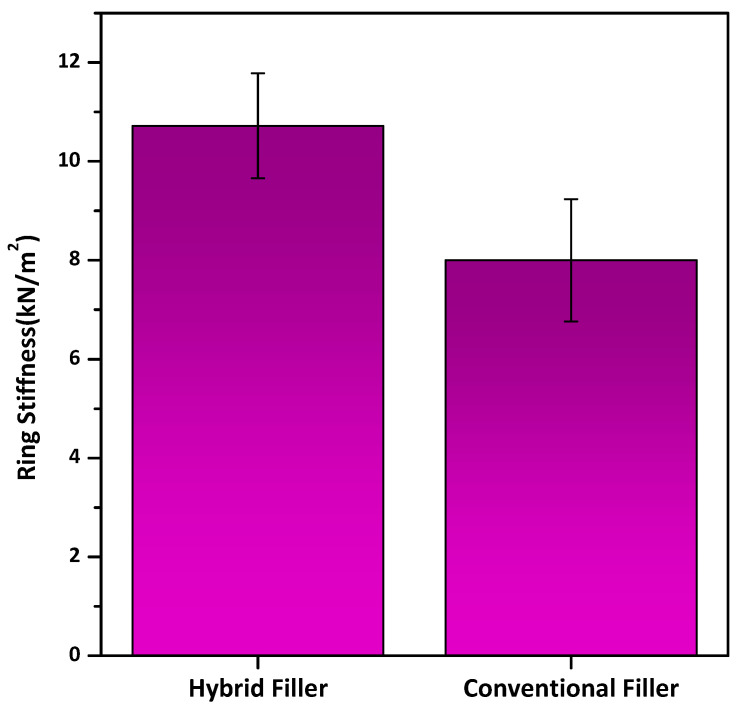
Comparison of the ring stiffness of double-wall corrugated pipes reinforced with POE-modified hybrid CaCO_3_/Talc filler versus conventional calcite filler.

The ring stiffness data demonstrates a clear enhancement in structural performance for the HDPE pipes containing the new filler formulation (Figure 18). The hybrid filler-added HDPE pipes exhibited ring stiffness values between 9.43 and 12.20 kN/m^−2^ (mean ~10.7 kN/m^−2^), whereas the standard filler-added pipes ranged from 6.12 to 9.06 kN m^−2^ (mean ~7.45 kN/m^−2^). This significant increment, corresponding to approximately 43%, is critical for corrugated pipes, as ring stiffness is the primary indicator of the pipe’s ability to resist vertical deflection under soil and traffic loads in underground applications.

Compared to conventional HDPE pipes filled solely with spherical calcium carbonate, the hybrid CaCO_3_–talc system demonstrates superior ring stiffness due to more efficient reinforcement and processing-induced structural stability. While CaCO_3_ increases stiffness mainly by restricting polymer chain mobility, its low aspect ratio limits deformation resistance. The incorporation of high-aspect-ratio talc platelets, which orient along the extrusion flow direction, provide enhanced resistance to deformation perpendicular to the pipe wall, directly improving ring stiffness. Although the formulation contains polyolefin elastomer (POE), its potential softening effect is offset by improved filler dispersion and interfacial adhesion promoted by the compatible HDPE carrier resin, enabling effective stress transfer. In addition, the optimized additive package stabilizes melt flow and wall thickness uniformity during corrugation, which further enhances load-bearing capacity, since ring stiffness depends on both material modulus and geometric consistency.

The present study advances the fundamental insights provided by Wang et al. regarding POE-modified calcite composites by scaling these concepts from laboratory specimens to full-scale industrial double-wall corrugated pipes [52]. While their work established that mineral loading enhances flexural modulus, our ring stiffness results validate this stiffening effect in a complex tubular geometry, with further enhancement attributed to the platy morphology of talc acting as a rigid skeletal framework, unlike the purely spherical calcite used in the reference study. Moreover, the toughening mechanism of POE, characterized by Wang et al. via impact strength improvements, is corroborated herein by the structural ductility observed in the ring flexibility analysis, confirming that the elastomeric phase effectively mitigates stress concentrations to prevent brittle failure during macroscopic deformation.

In summary, an inorganic filler system of calcite and talc modified with POE was developed to improve HDPE corrugated pipe performance. The compound, containing erucamide, PE wax, montan wax, and PIB, showed homogeneous dispersion and stability. Filler addition increased density and MFI maintained OIT up to 40 min, and enhanced ring stiffness to 12.20 kN/m^2^. The system exhibited uniform filler dispersion and thermal stability, ensuring smooth extrusion under industrial conditions. Overall, the designed filler structure demonstrates strong potential for scalable application in high performance HDPE double-wall corrugated pipe manufacturing.

## 4. Conclusions

This study successfully engineered a POE-modified hybrid CaCO_3_-Talc filler system as a high-performance alternative to conventional calcite formulations for infrastructure-grade HDPE pipes. Comprehensive characterization (XRD, XRF, FTIR, and SEM-EDX) confirmed the targeted chemical composition and a homogeneous phase distribution within the double-wall corrugated pipe matrix. Thermal analysis revealed a stable, component-resolved decomposition profile, with organic phases degrading between 455.1 °C and 484 °C. Notably, the hybrid filler formulation increased the average ring stiffness from 7.45 to 10.7 kN/m^2^ (~43%) compared to conventional calcite-filled pipes, while maintaining ring flexibility. This improvement is attributed to the synergistic effect of rigid mineral fillers and the POE phase, which mitigated mineral-induced embrittlement. The POE effectively mitigated mineral-induced embrittlement, a common failure mode in highly filled polyolefins. Oxidation Induction Time (OIT) was maintained above 20 min, while optimized rheological behavior facilitated stable extrusion and reduced surface defects.

While this provides significant short-term mechanical and processing advantages, further research is required to quantify long-term durability. Future efforts should focus on standardized hydrostatic pressure testing, slow crack growth (SCG) resistance, and molecular-level modeling of the POE–mineral interface to establish the long-term reliability of these hybrid systems in field applications. Furthermore, systematic investigations of varying additive compositions within the hybrid filler system, specifically by optimizing the ratios of mineral reinforcements to functional processing aids, are planned to tailor the composite’s performance for even more specialized infrastructure requirements.

## Figures and Tables

**Table 1:** Material specifications.

Material	Company	Density (g/cm^3^)	Avg. Particle Size (d_50,_ μm)	Purity (%)	MFI (g/10 min)
Calcite (Ca(CO_3_)	Niğtaş 3K (Niğde, Türkiye)	2.71	3.3	99	-
Talc (Mg_3_Si_4_O_10_(OH)_2_)	Niğtaş 5X (Niğde, Türkiye)	2.75	4.1	99–100	-
HDPE	NCT 5502 BL (İzmir, Türkiye)	0.954	-	-	1.2
POE	Exxonmobile Exact 5171 (Houston, TX, USA)	0.868	-	-	1

**Table 2:** Wall thickness values of the ∅200 HDPE double-wall corrugated pipes.

	ISO 13476-3	Double-Wall Corrugated Testing Pipes		
e_4min_ (mm)	1.5	2.24	2.50	2.70
e_5min_ (mm)	1.1	1.37	1.65	1.93

**Table 3:** The bulk composition (wt.%) of the studied filler, measured by XRF analysis.

Component	Result	Unit	Det. Limit (ppm)
CaO	66.0144	wt.%	0.01056
SiO_2_	17.5543	wt.%	0.01232
MgO	12.4189	wt.%	0.01793
Al_2_O_3_	0.9010	wt.%	0.01148
Na_2_O	0.6451	wt.%	0.01389
SO_3_	0.1142	wt.%	0.00282
K_2_O	0.1705	wt.%	0.00239
MnO	0.0479	wt.%	0.00633
Fe_2_O_3_	1.9584	wt.%	0.04040
Cr_2_O_3_	0.0690	wt.%	0.00935
P_2_O_5_	0.0427	wt.%	0.00222
TiO_2_	0.0638	wt.%	0.01688

**Table 4:** Results of quantitative mineralogical analysis.

	Calcite Ca(CO_3_)	Talc Mg_3_Si_4_O_10_(OH)_2_	Kaolinite Al_2_Si_2_O_5_(OH)_4_
The Filler	76.8%	21.7%	1.5%

**Table 5:** Main FT-IR absorption bands of the composite and their assignments according to the literature.

Wavenumber (cm^−1^)	Assignment (Vibration)	Component
3670–3600	O-H stretching (structural hydroxyl)	Talc (Mg_3_Si_4_O_10_(OH)_2_), surface OH groups
2916–2848	CH_2_ asymmetric/symmetric stretching	HDPE matrix, polyolefin elastomer, erucamide, montan wax, PE wax, PIB
~1795	C=O stretching/overtone (carbonyl functionality)	Erucamide, montan wax (fatty acid amide/ester moieties), minor oxidation
~1410	CH_2_ bending + CO_3_^2−^ asymmetric stretching	Polyolefin chains + calcite (CaCO_3_)
1010	Si-O stretching	Talc
872	CO_3_^2−^ out-of-plane bending	Calcite (CaCO_3_)
712	CO_3_^2−^ in-plane bending	Calcite (CaCO_3_)
686	Si-O/Mg-O lattice vibrations	Talc
729–720	CH_2_ rocking	HDPE matrix, polyolefin elastomer
542	Si-O-Mg deformation/lattice modes	Talc

## Data Availability

The original contributions presented in this study are included in the article. Further inquiries can be directed to the corresponding authors.

## Appendix A. Characterization of Neat HDPE Reference Material

This section provides the comprehensive structural, thermal, and morphological characterization of the high-density polyethylene (HDPE) used as the matrix material in this study. The Neat HDPE was analyzed to establish a baseline for comparison with the hybrid-filled composites.

### Appendix A.1. Morphological and Elemental Analysis (SEM-EDX)

Morphology and elemental composition of the Neat HDPE were investigated using SEM coupled with EDX, shown in Figure A1 and Figure A2, respectively.

### Appendix A.2. Thermal Gravimetric Analysis (TGA/DTG)

The thermal stability of the Neat HDPE was evaluated under a nitrogen atmosphere from 30 °C to 700 °C. As shown in Figure A3, the material exhibits a single-step degradation profile, characteristic of high-purity polyolefins.

### Appendix A.3. Fourier Transform Infrared Spectroscopy (FTIR)

The chemical structure of the Neat HDPE was confirmed via FTIR spectroscopy (Figure A4).

The chemical and structural integrity of the Neat high-density polyethylene (HDPE) used as the polymer matrix was verified through Fourier Transform Infrared (FTIR) spectroscopy. The reference spectrum, provided in Figure A4, confirms the high purity and characteristic molecular structure of the material.
